# Extracellular acidification leads to mitochondrial depolarization with following free radical formation in rat brain synaptosomes

**DOI:** 10.1186/2193-1801-4-S1-P12

**Published:** 2015-06-12

**Authors:** Sergei Fedorovich, Tatyana Pekun, Sviatlana Hrynevich, Tatyana Waseem

**Affiliations:** Institute of Biophysics and Cell Engineering NASB, Belarus

**Keywords:** synaptosomes, ischemia, free radicals

Brain ischemia is accompanied by lowering of pHo and pHi. We investigated an influence of of acidosis on free radical formation in synaptosomes. Three models were used.

1) Strong extracellular acidification down to pHo 6.0.

2) Moderate extracellular acidification down to pHo 7.0

3) Intracellular acidification induced by addition of 1 mM amiloride corresponding to pHi decrease down to 6.65.

We have shown that both types of extracellular acidification, but not intracellular acidification, increase DCFDA fluorescence by calcium-independent way that reflects free radical formation. These three treatments induce the rise of the dihydroethidium fluorescence that reports synthesis of superoxide anion. However, the impact of low pHi on superoxide anion synthesis was less than induced by moderate extracellular acidification. Mitochondrial uncoupler CCCP did not inhibit an increase of fluorescence of both dyes at pHo 6.0. In contrast, superoxide anion synthesis at pHo 7.0 was almost completely eliminated by CCCP. Furthermore, using fluorescent dyes JC-1 and rhodamine-123, we confirmed that decrease of pHo leads to mitochondria depolarization. Low pHi was not effective. Iron chelator deferoxamine and antioxidant ionol are inhibits pH-induced increase of DCFDA fluorescence, but does not influenced mitochondria depolarization. We are failed to found sodium influx monitored by fluorescent dye Sodium Green in case of low pHo. Involving of plasma membrane receptor which is distinct from acid-sensitive ion channels (ASIC) and electron transport chain of mitochondria for moderate acidification can be suggested. Action of strong acidification seems to be mediated by release of iron from proteins. We have shown that low pHo led to oxidative stress in neuronal presynaptic endings that might underlie the long term irreversible changing in synaptic transmission.

